# Genetic modification of *Streptococcus dysgalactiae* by natural transformation

**DOI:** 10.1128/msphere.00214-24

**Published:** 2024-06-21

**Authors:** Marita Torrissen Mårli, Oddvar Oppegaard, Davide Porcellato, Daniel Straume, Morten Kjos

**Affiliations:** 1 Faculty of Chemistry, Biotechnology and Food Science, Norwegian University of Life Sciences, Ås, Norway; 2 Haukeland University Hospital, University of Bergen, Bergen, Norway; The University of Arizona, Tucson, Arizona, USA

**Keywords:** natural transformation, pyogenic streptococci, genetic tools, genetic competence

## Abstract

*Streptococcus dysgalactiae* is an emerging human and animal pathogen. Functional studies of genes involved in virulence of *S. dysgalactiae* and other pyogenic group streptococci are often hampered by limited genetic tractability. It is known that pyogenic streptococci carry genes required for competence for natural transformation; however, in contrast to other streptococcal subgroups, there is limited evidence for gene transfer by natural transformation in these bacteria. In this study, we systematically assessed the genomes of 179 *S*. *dysgalactiae* strains of both human and animal origins (subsp. *equisimilis* and *dysgalactiae,* respectively) for the presence of genes required for natural transformation. While a considerable fraction of the strains contained inactive genes, the majority (64.2%) of the strains had an intact gene set. In selected strains, we examined the dynamics of competence activation after addition of competence-inducing pheromones using transcriptional reporter assays and exploratory RNA-seq. Based on these findings, we were able to establish a protocol allowing us to utilize natural transformation to construct deletion mutants by allelic exchange in several *S. dysgalactiae* strains of both subspecies. As part of the work, we deleted putative lactose utilization genes to study their role in growth on lactose. The data presented here provide new knowledge on the potential of horizonal gene transfer by natural transformation in *S. dysgalactiae* and, importantly, demonstrates the possibility to exploit natural transformation for genetic engineering in these bacteria.

**IMPORTANCE:**

Numerous *Streptococcus* spp. exchange genes horizontally through natural transformation, which also facilitates efficient genetic engineering in these organisms. However, for the pyogenic group of streptococci, including the emerging pathogen *Streptococcus dysgalactiae*, there is limited experimental evidence for natural transformation. In this study, we demonstrate that natural transformation *in vitro* indeed is possible in *S. dysgalactiae* strains under optimal conditions. We utilized this method to perform gene deletion through allelic exchange in several strains, thereby paving the way for more efficient gene engineering methods in pyogenic streptococci.

## INTRODUCTION

*Streptococcus dysgalactiae*, an emerging animal and human pathogen, is phylogenetically divided into two distinct subspecies: *Streptococcus dysgalactiae* subsp. *dysgalactiae* (SDSD) and *Streptococcus dysgalactiae* subps. *equisimilis* (SDSE) (1). SDSE is predominantly recognized for its role in human disease, contributing to a range of infections including superficial skin infections, streptococcal toxic shock syndrome, meningitis, and endocarditis (1). On the other hand, SDSD affects animals, particularly cattle, where it is a leading cause of bovine mastitis (2). *S. dysgalactiae* is classified within the pyogenic group and is a close relative to the human pathogen *Streptococcus pyogenes*. Functional studies of genes in these species are often hampered by their poor genetic tractability. Gene modifications in these species are mostly done using temperature sensitive vectors (3–5), a time-consuming process, which is challenged by the presence of type I restriction modification systems targeting DNA with foreign methylation patterns (6, 7).

Natural genetic transformation, a process in which bacteria take up exogenous DNA and incorporate it into their genomes by homologous recombination, is a key mechanism for evolution, adaptation, and spread of antibiotic resistance in bacteria (8). Many streptococcal species outside the pyogenic subgroup have been shown to be naturally transformable, even *in vitro* under laboratory conditions. Most prominently, this includes not only *Streptococcus pneumoniae*, which has long served as a model for studies of natural transformation, but also other species such as *Streptococcus mitis*, *Streptococcus gordonii*, *Streptococcus anginosus*, *Streptococcus mutans*, *Streptococcus ferus, Streptococcus thermophilus*, *Streptococcus salivarius*, *Streptococcus suis*, *Streptococcus vestibularis*, and *Streptococcus infantarius* (9–19). The ability to perform *in vitro* transformation makes it possible to harness natural transformation as a tool for genetic engineering in these organisms, greatly aiding gene function studies, giving us deeper insight into the factors that govern pathogenicity, antimicrobial resistance mechanisms, and other key features of these species.

**Fig 1:**
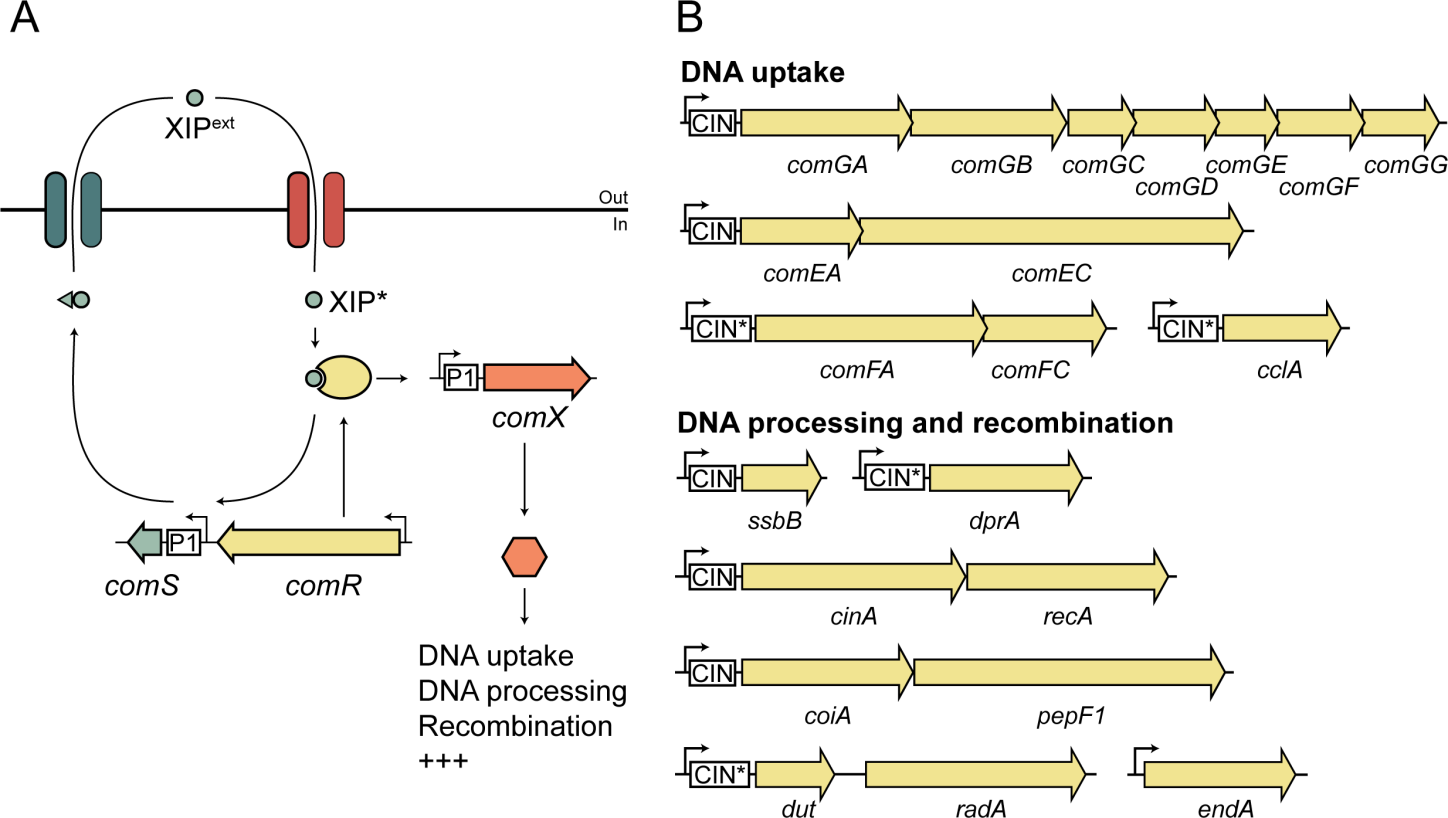
(**A**) Schematic representation of the ComRS competence regulatory system in *S. dysgalactiae*. ComS is exported and processed into its mature form (XIP), which is subsequently reimported into the cell via the Opp/Ami transporter. Once inside, XIP binds to the ComR transcriptional regulator, forming the XIP-ComR complex, which in turn activates transcription from the P1 promoter of *comS* and *comX/sigX* (consensus sequence for P1 in *S. dysgalactiae* is AANAGGACAN_3_ATGTCNTNTTNNTN_16_TATANT [22]). The alternative sigma factor ComX induces transcription of late competence genes needed for uptake and integration of DNA. (**B**) Diagram of the operon structure of core competence genes known to be required for natural transformation in streptococci harboring the *comRS* system. These genes are implicated in different steps of DNA uptake and transport, as well as in DNA processing and recombination. Binding sites for ComX, known as CIN-boxes (CIN), with consensus sequence TACGAATA, are shown. Variants of the consensus CIN-box sequence that differ in the first two bases are marked with an asterisk.

Central to natural transformation is a finely tuned regulatory network controlling the so-called competent state, i.e., the state in which the uptake and integration of foreign DNA can occur. There are two systems known to regulate competence in streptococci: the ComCDE system present in the mitis and anginosus group streptococci (including *S. pneumoniae*), and the ComRS system characteristic of the pyogenic, mutans, bovis, suis and salivarius group streptococci (20). *S. dysgalactiae*, a member of the pyogenic group, encodes the ComRS regulatory system. In this system (Fig. 1), which has been most extensively studied in the mutans and salivarius groups, a peptide precursor ComS is produced and processed upon export to produce the mature quorum sensing XIP peptide pheromone (20, 21). XIP is imported into the cytoplasm via the Opp/AmiA oligopeptide permease, where it interacts with the peptide-binding tetratricopeptide repeat (TPR) domain of an Rgg-like transcriptional activator, ComR, which in turn changes conformation and binds to consensus sequences in the promoter region (termed P1 or Ecom-box), triggering a positive feedback loop resulting in increased expression of ComS and the alternative sigma factor ComX (also known as SigX) (20–24). ComX specifically recognizes and binds to conserved DNA sequences, termed the CIN-boxes, located upstream of the late competence genes involved in DNA uptake and recombination. Together, this activates the ComX regulon and the competent state. The factors required for DNA uptake include a DNA-binding type IV-like pseudopilus encoded by the *comG* operon and a DNA-binding protein, ComEA, which guides the DNA to the transmembrane protein ComEC (25). After cleavage of one of the DNA duplex strands by the nuclease EndA, single-stranded DNA (ssDNA) is passed through ComEC with the help of ComFA. Once inside the cell, the ssDNA is protected from nucleases by the ssDNA-binding proteins SsbB and DprA. Subsequently, the homology-dependent recombinase, RecA, is loaded on the DNA, allowing for identification of homology regions and homologous recombination to insert the foreign DNA into the genome. While most of these genes are thought to be competence induced, the system is also dependent on non-competence-regulated genes, such as the Opp peptide transporters and the constitutively expressed endonuclease EndA (26).

In a systematic study of the competence regulon in *S. pyogenes*, it was noted that despite harboring all the genes needed for natural transformation, a number of the strains analyzed had mutations in the genes encoding the DNA uptake system (23). A high incidence of transposable elements in the vicinity of genes involved in competence in *S. pyogenes* has also been reported (27). Additionally, a prophage-encoded protein known as paratox has been shown to inhibit competence induction by preventing the interaction between XIP and ComR in *S. pyogenes* (28, 29). Notably, even in strains with a complete set of competence genes, no genetic transformation has been observed *in vitro*, although the genes were transcriptionally activated by the addition of XIP peptides (23). This result was attributed to lack of efficient DNA uptake (23). Interestingly, however, natural transformation in *S. pyogenes* was shown to occur experimentally under biofilm conditions during murine *in vivo* colonization (30).

*S. dysgalactiae* harbors a competence system similar to *S. pyogenes* (20, 23), but natural transformation has not been extensively studied in this species. In this work, we identified SDSD and SDSE strains with a set of intact genes required for competence induction and natural transformation, and using transcription assays as a guide, we demonstrate that genetic engineering by utilizing the natural transformation process is possible in this species.

## RESULTS

### Presence of early and late competence genes in *S. dysgalactiae* suggests potential for natural transformation

**Fig 2:**
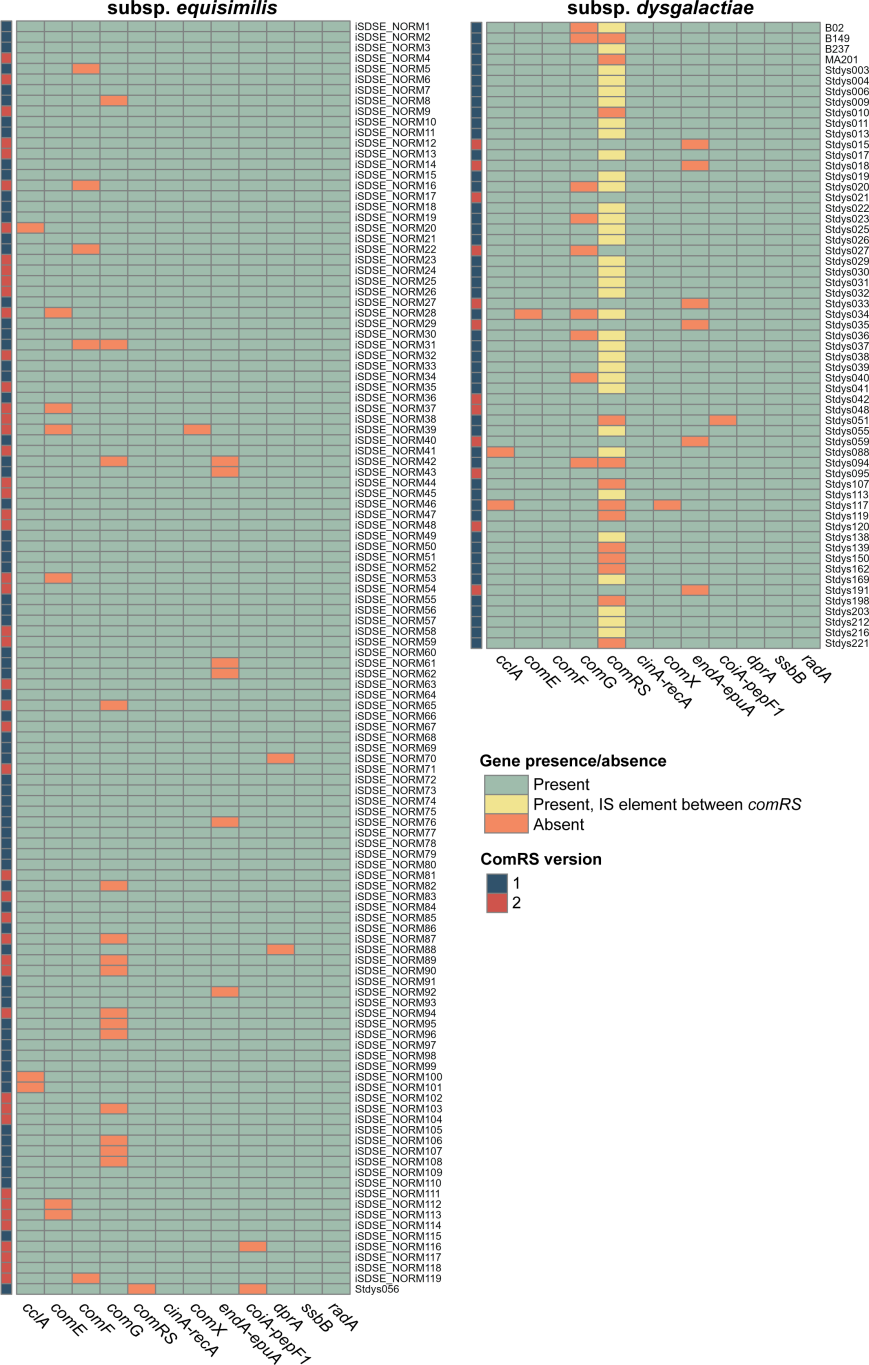
Competence genes in *S. dysgalactiae*. Heatmap showing the presence of intact genes/operons required for competence for natural transformation (25, 31) within strains of *S. dysgalactiae* subsp. *equisimilis* and *S. dysgalactiae* subsp. *dysgalactiae*. See also Fig. 1 and Table 1 for overview of genes. Green indicates the presence of an intact gene; orange denotes inactive genes (due to mutation or insertion of prophage or mobile element); and yellow shows the presence of an insertion sequence (IS) between the *comR* and *comS* genes (see Table S1 for details). The *comE*-operon (*comEA-EC*), *comF*-operon (*comFA-FC*), and *comG*-operon (*comGA-GC-GD-GE-GF-GG*) are each indicated by a single column. The *comRS* allele in each strain is indicated by color coding in the leftmost column, with dark blue for *comRS1* and red for *comRS2*.

To our knowledge, natural transformation in pyogenic streptococci has not been observed under *in vitro* conditions. To analyze the potential of natural transformation in *S. dysgalactiae*, the presence and intactness of genes supposedly needed for competence and natural transformation (Fig. 1A and B) were analyzed in a collection of 59 SDSD and 120 SDSE isolates derived from animal and human collections in Norway. The analysis revealed that 54.2% (32 of 59) of SDSD isolates and 69.2% (83 of 120) of SDSE isolates harbor a complete set of intact genes necessary to perform natural transformation (Fig. 2; Table S1). The remaining strains typically had disruptions in key genes such as *comR*, *comX*, *cclA*, *comGD*, *comGF*, *comEC*, *comFA*, *comFC*, *endA*, *coiA*, or *dprA* (Fig. 2; Table S1). These disruptions were attributed to truncations as a result of mutations, prophages, or mobile insertion elements (Table S1). Notably, 57.6% of SDSD isolates (34 of 59 isolates) contained an insertion sequence (IS) element inserted between the *comR* and *comS* genes, without interrupting the open reading frames, while 33.3% of SDSEs (40 of 120 isolates) harbored an IS element downstream the *comS* gene.

**TABLE 1:** Expression core competence genes in *S. dysgalactiae* strains iSDSE-NORM77 and Stdys021^*a*^*^,^*^*b*^

	Gene	iSDSE-NORM77			Stdys021		
		TPM 0 min	L2FC 10 min	L2FC 60 min	TPM 0 min	L2FC 5 min	L2FC 30 min
Regulation	*comR*	27.93	0.07	**3.38**	11.41	0.07	0.66
	*comS*	25.45	**1.73**	**3.59**	30.57	**2.81**	**2.92**
	*comX*	7.47	**4.9**	**6.59**	72.77	0.87	0.89
DNA uptake	*comGA*	6.78	**4.18**	**6.82**	9.03	**3.37**	**4.68**
	*comGB*	3.6	**5.5**	**8.13**	6.14	**4.3**	**5.79**
	*comGC*	3.5	**5.79**	**8.36**	4.25	**4.95**	**6.41**
	*comGD*	3.27	**6.15**	**8.66**	4.61	**5.01**	**6.48**
	*comGE*	1.64	**7.25**	**9.74**	4.09	**5.3**	**6.77**
	*comGF*	2.73	**6.53**	**9.02**	4.88	**4.96**	**6.42**
	*comGG*	5.35	**5.64**	**8.14**	7.18	**4.45**	**5.95**
	*comEA*	5.66	**4.18**	**6.90**	1.99	**4.65**	**5.84**
	*comEC*	2.26	**5.36**	**8.07**	2.12	**4.24**	**5.65**
	*comFA*	5.83	**3.72**	**6.12**	1.58	**3.90**	**5.00**
	*comFC*	7.22	**3.4**	**5.71**	1.9	**3.81**	**4.85**
	*cclA*	16.84	**1.07**	**2.98**	4.01	**1.86**	**3.2**
DNA processing and recombination	*dprA*	7.24	**4.75**	**6.85**	3.48	**3.88**	**5.28**
	*ssbB*	34.71	**3.92**	**6.29**	15.31	**5.09**	**6.31**
	*cinA*	35.21	**2.35**	**4.66**	105.47	0.71	**2.22**
	*recA*	686.39	0.25	**1.13**	841.13	−0.09	−0.26
	*coiA*	27.68	**4.05**	**6.24**	30.23	**1.58**	**2.67**
	*pepF1*	215.76	**1.56**	**3.28**	352.35	0.2	0.81
	*radA*	209.85	0.32	0.67	325.66	−0.15	−0.33
	*endA*	159.92	0.1	−0.13	112.93	−0.17	−0.31

^*a*^ Boldface data indicate increased expression upon XIP treatment (L2FC, log_2_ fold change > 1).

^*b*^ TPM, transcript per million.

Furthermore, our genomic analysis showed, in line with previous studies (21), that *S. dysgalactiae* has two allelic forms of *comS* (named *comS1* and *comS2*) and *comR* (encoding ComR1 and ComR2) in both subspecies. The *comS1* allele was most prevalent, found in 79.7% of SDSD isolates (47 of 59 isolates) and in 60% of SDSE isolates (72 of 120 isolates) (Fig. 2). The encoded ComS1 and ComS2 precursor peptides of 32 and 31 amino acids, respectively, both feature a double tryptophan (WW) motif within the mature XIP peptide sequence (XIP1: EFDWWNLG and XIP2: QVDWWRL), and these peptides recognize and bind the cognate ComR protein. The presence of conserved ComX binding sites (CIN-boxes) were identified in the promoter regions of strains Stdys021 and iSDSE-NORM77. The consensus sequence TACGAATA was identified in the promoter regions upstream of *comGA*, *comEA*, *ssbB*, *cinA*, and *coiA*, while some genes had a sequence varying slightly from the consensus, namely, *comFA* (GTCGAATA), *cclA* (TTCGAATA), *dprA* (TTCGAATA), and *dut* (TGCGAATA) (Fig. 1B) (23). These findings together indicate that the underlying genetic machinery for natural transformation is indeed present in a majority of the *S. dysgalactiae* strains.

### The master competence regulator ComX and the late competence genes are induced by XIP in *S. dysgalactiae*

**Fig 3:**
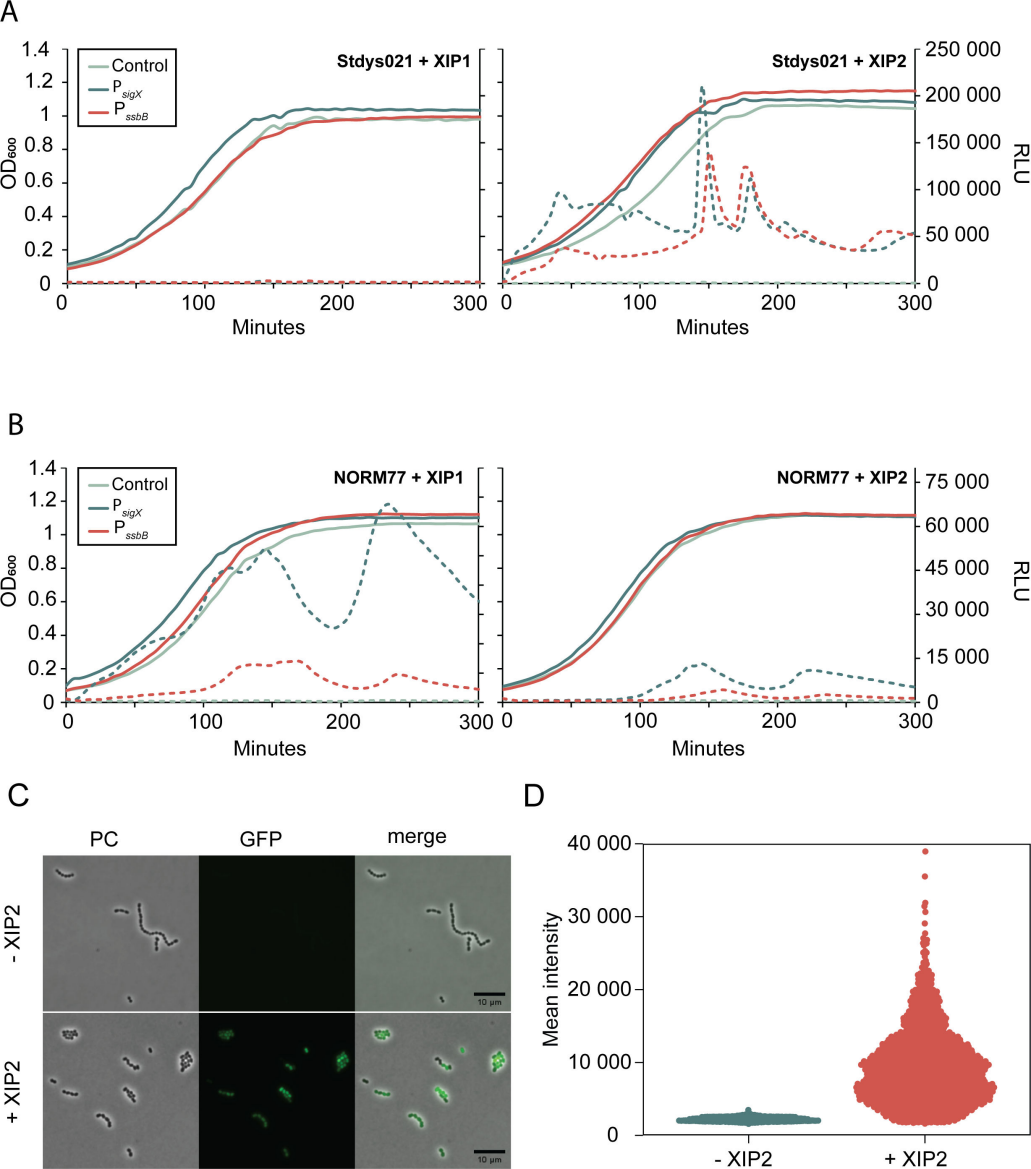
Competence reporter assays. Luciferase expression from the P*_comX_*-*luc*-*gfp* and P*_ssbB_*-*luc*-*gfp* reporters in Stdys021 (**A**) and iSDSE-NORM77 (**B**) after addition of XIP1 or XIP2. Cells were diluted to an OD_600_ of 0.01 and grown at 37°C until an OD_600_ of 0.1 before 250-ng/mL XIP was added. OD_600_ and luminescence (as relative luminescence units [RLUs]) were measured every 5 min. Control strain harbors the pFD116 vector. Solid lines indicate growth (OD_600_), while dashed lines correspond to RLU. Data shown are representative of three experiments (**C**) Microscopy of Stdys021 reporter strain P*_comX_*-*luc*-*gfp* with or without XIP2 induction. Phase contrast (PC), GFP images, and overlays are shown. The cells were diluted to an OD_600_ of 0.01 and grown at 37°C until an OD_600_ of 0.1 before 250-ng/mL XIP2 was added. Samples were taken for microscopy 1 hour post-induction. Micrographs are representative of three technical replicates. The scale bar is 10 µm. (**D**) Swarm plot showing quantitative analysis of microscopy assay. Each dot represents mean GFP intensity per cell (*n* = 2,771 cells). Mean GFP signals per cell with or without XIP2 induction are plotted. Styds021 harboring P*_comX_*-*luc*-*gfp* reporter plasmid was diluted to an OD_600_ of 0.01 and grown at 37°C until an OD_600_ of 0.1, at which time 250-ng/mL XIP2 was added.

Based on the genome analysis (Fig. 2), we selected two strains for further analyses, both harboring a complete and intact set of genes needed for natural transformation: the SDSD strain Stdys021 and the SDSE strain iSDSE-NORM77. Neither of these strains has the paratox gene reported to inhibit competence development in *S. pyogenes* (28) (Table S1). To investigate the competence activation in these strains, we constructed transcriptional reporter plasmids with firefly luciferase (*luc*) and GFP (*gfp*) under the control of an early (*comX*) and a late *(ssbB*) competence gene promoter. These reporter plasmids were electrotransformed into the selected strains, and growth (OD_600_) and luminescence were monitored over time. In the absence of XIP induction, baseline expression from the *comX* and *ssbB* promoters remained low in both strains, suggesting low or no autoinduction of competence under these conditions (Fig. 3A and B). Upon addition of the cognate XIP peptides (XIP2 for Stdys021 and XIP1 for NORM77), we observed quick induction of the *comX* promoter in both strains, with Stdys021 responding even faster than iSDSE-NORM77 (Fig. 3A and B). Interestingly, the expression pattern from the *comX*-promoter appeared to be variable during growth under these conditions, as observed by the fluctuating luminescence values post-induction (Fig. 3A and B). Notably, there were three distinct peaks of P*_comX_* activation: one during early log phase, one at the transition from exponential to stationary phase, and one within the stationary phase. Expression from the *ssbB* promoter mirrored the *comX* expression pattern in both strains, although with a shifted response and generally lower signal (Fig. 3A and B).

The potential cross-activity between the two pheromone variants was also explored by adding the non-cognate XIP to the reporter strains (XIP1 to Stdys021 and XIP2 to iSDSE-NORM77). In Stdys021, addition of XIP1 resulted in only a marginal increase in expression, just above the baseline of the uninduced condition (Fig. 3A). On the other hand, addition of XIP2 in iSDSE-NORM77 did result in increased *comX* and *ssbB* expression (Fig. 3B), although the response to XIP2 was delayed compared to XIP1, with the signal intensity approximately fourfold lower. These observations in iSDSE-NORM77 are in agreement with previous findings where SDSE demonstrated capacity to respond to a wide range of non-cognate XIPs when tested in a heterologous system, suggesting that the peptide-binding domain of this ComR is rather promiscuous (20).

In order to see whether the induction was uniform across the cell population, we performed fluorescence microscopy on Stdys021 harboring the P*_comX_*-*luc*-*gfp* reporter plasmid. The micrographs and quantitative analysis of the mean GFP intensity per cell confirmed that, upon induction with XIP2, P*_comX_* indeed was active in 90.4% of the cells (defined as having a fluorescence signal above the maximum fluorescence signal observed in the negative control condition; Fig. 3C), confirming robust competence induction. However, analysis at the single-cell level highlighted heterogeneity in expression within the population, as depicted by the swarmplot in Fig. 3D, which showed varying GFP intensities among individual cells. A minor subset of cells (9.6%) had GFP signals at levels comparable to the uninduced control, underscoring a non-uniform response to induction.

### The competence regulon in *S. dysgalactiae* is induced by exogeneous XIP

To gain a deeper understanding of the competence regulon in *S. dysgalactiae* and to check whether all the genes required for natural transformation were expressed, we performed an explorative, time-resolved RNA-seq analysis on iSDSE-NORM77 (SDSE) and Stdys021 (SDSD). We compared samples pre- and post-XIP induction at two time points chosen based on the induction of competence observed in the reporter strains (Fig. 2): 10 and 60 min for iSDSE-NORM77, and 5 and 30 min for Stdys021.

**Fig 4:**
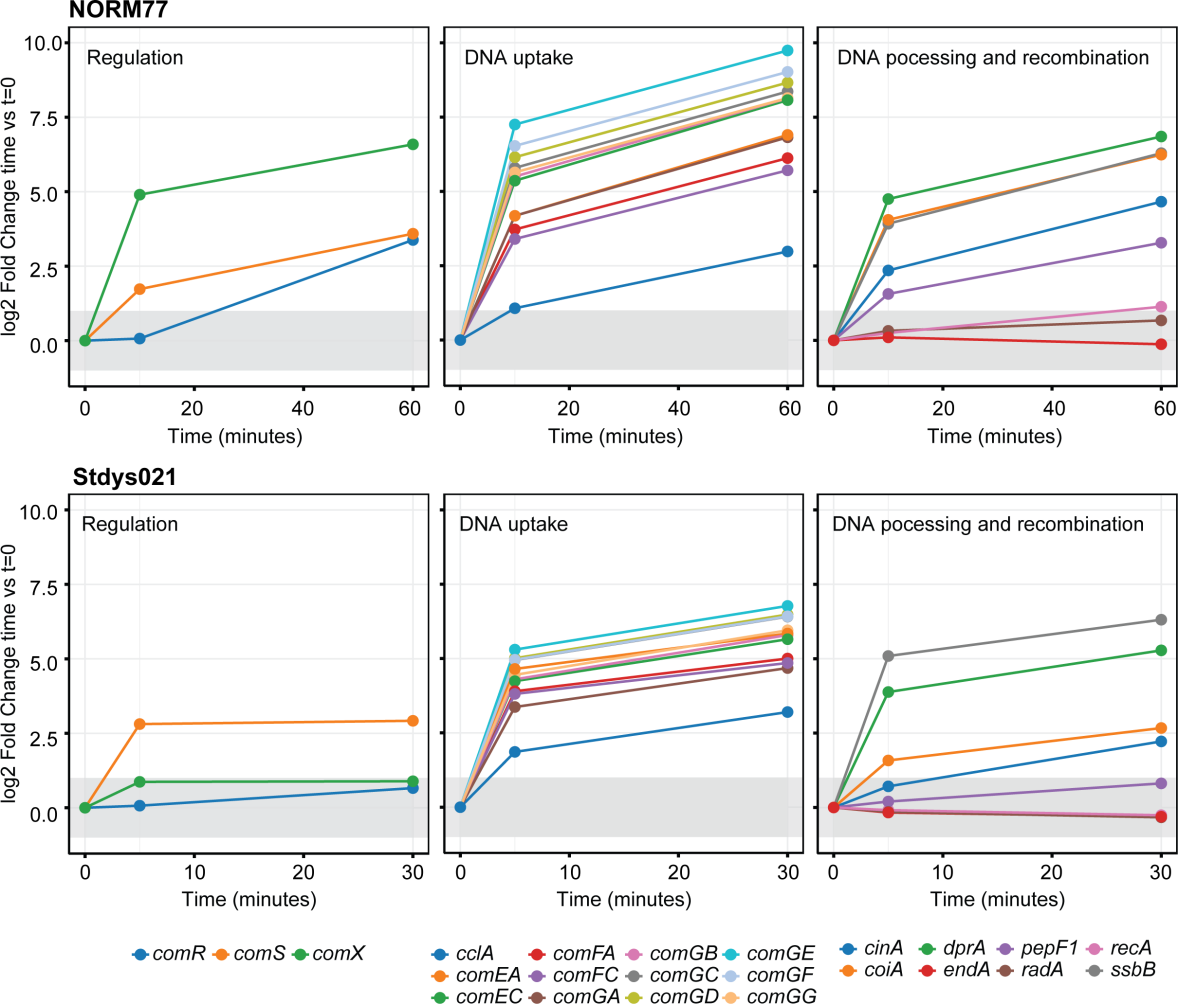
RNA-seq expression profiles. Expression profiles of genes involved in competence and natural transformation upon induction of competence by cognate XIP in iSDSE-NORM77 and Stdys021. Genes involved in competence regulation, DNA uptake, and DNA processing and recombination are plotted separately. Each line shows the log_2_ fold changes per time point versus *t* = 0 (before XIP addition) for each gene. The gray-shaded area corresponds to a log2 fold change of −1 to 1, and expression within this area is not considered to be differentially expressed.

The transcription profiles revealed that all genes involved in DNA uptake, DNA processing, and homologous recombination were either upregulated (*comGA-comGG*, *comEA-comEC*, *comFA-comFC*, *cclA*, *dprA*, *ssbB*, *cinA*, and *coiA*) upon XIP induction or constitutively highly expressed (*recA*, *pepF1*, *radA*, and *endA*) in both strains (Table 1). The upregulation was characterized by an increase in expression at the first time point followed by sustained expression throughout the experiment (Table 1; Fig. 4; Tables S2 to S6). For the competence regulatory genes (*comR*, *comS*, and *comX*), some variability in expression was observed between the strains. For iSDSE-NORM77, expression levels were induced for both *comS* and *comX* at the first time point, with expression levels increasing further at 60 min, while expression of *comR* was upregulated only at 60 min post-induction (Table 1; Fig. 4). In Stdys021, an increase in expression was detected only for *comS* (Table 1; Fig. 4), while *comX* surprisingly appeared to have a relatively high expression already at *t* = 0 under these conditions (Table 1; Tables S2 to S6). This differs from what was observed in the reporter assay in which expression from P*_comX_* was clearly induced (Fig. 3B). Despite this non-coherent regulation of the early competence genes under these conditions, we conclude that the components needed for natural transformation are active after XIP induction in *S. dysgalactiae* Stdys021 and iSDSE-NORM77.

Beyond the core competence genes which expectedly displayed the highest log_2_FC, the transcription data revealed that with |log_2_FC > 1| as threshold, there was differential expression of 140 genes (7.5% of the genes, 79 genes upregulated, 61 genes downregulated) in Stdys021 and 252 genes (12.4% of the genes, 156 genes upregulated, 95 genes downregulated) in iSDSE-NORM77 at one or both time points post-induction (Tables S2 to S6; Fig. S1A and B). The lack of robust statistics (two replicates were analyzed for Stdys021 and one for iSDSE-NORM77) precludes firm conclusions of the regulation of genes with low log_2_FC, but a comparison between the strains revealed that 42 genes were upregulated at one or both time points in both strains, while 12 genes were downregulated at one or both time points in both strains (Tables S5 and S6). It should also be noted that most of these differentially regulated genes were only differentially regulated at the last time point, suggesting indirect regulation and not a direct effect by the addition of XIP. The genes regulated at the first time point included one encoding an SH3 domain containing putative murine hydrolase and one gene encoding a lipid II:glycine glycyltransferase (*femX*). A CIN-box was found in the promoters of both genes, which are probably directly regulated by ComX. Furthermore, a slight upregulation of the HrcA stress-response regulon, which includes major chaperones involved in the bacterial heat-shock response (Tables S5 and S6), was observed at the first time point in both strains. Similar observations have been made in *S. pneumoniae*, where competence induction correlates with the upregulation of stress-related genes, including those in the HrcA regulon (26, 32–34). Surprisingly, we also noted that genes *adhE*, *adhA*, and *pflB*, which encode proteins involved in fermentative metabolism by converting pyruvate to ethanol and formate, were upregulated in both strains. To understand whether this upregulation was a direct consequence of competence induction or resulted from an indirect effect of the experimental conditions, the concentrations of metabolites (glucose, pyruvate, lactate, acetate, DL-pyroglutamate, acetaldehyde, and ethanol) were compared between non-induced and XIP-induced cells using high-performance liquid chromatography (HPLC) (Fig. S2A) and head space gas chromatography (HSGC) (Fig. S2B). These analyses revealed that there were no differences between XIP-induced and non-induced cells, suggesting that the observed shift in expression of metabolic genes is more likely attributable to changes in the growth phase between *t* = 0 and *t* = 30/60, rather than being a direct consequence of competence induction.

### Allelic exchange in SDSD and SDSE by *in vitro* natural transformation

Given that the competence regulon in *S. dysgalactiae* was successfully activated by XIP addition, we tested whether natural transformation could be used to make gene knockouts in this species. In an attempt to knock out the β-galactosidase gene *lacZ* in Stdys021, a putative non-essential gene under these growth conditions, a PCR product comprising a kanamycin resistance marker flanked by the 2-kb homology regions of *lacZ* was generated. Long (2 kb) homology flanking regions have been shown to provide higher transformation rates in other streptococci (10, 13). Initially, we tested conditions similar to those used in our RNA-seq experiments to obtain a transformation protocol. In attempts to improve transformation rates, we tested various modifications, including adjusting the OD_600_ at which competence was induced with XIP (ranging from OD_600_ 0.05–0.6), the timing of DNA addition after inducing to competence (0, 2, or 3 hours post-induction), and the duration of incubation post-DNA addition (2, 3, and 4 hours and overnight). We also varied the concentration of the XIP peptide used to induce competence (up to 1,000 ng/mL). In the final protocol, overnight cultures were first diluted in C-medium to an initial OD_600_ of 0.05. These cultures were then incubated until reaching an OD_600_ of 0.1–0.2, ensuring that cells were actively dividing. Following this initial growth phase, the cultures were further diluted to an OD_600_ of 0.02–0.03 and then grown again to an OD_600_ of 0.05. Then, the cells were induced with 250 ng/mL XIP, and 500 ng of DNA was added. The cultures were incubated for 3–4 hours at 37°C prior to selective plating. Using this optimized protocol, we were able to successfully generate gene deletions by allelic replacement (Δ*lacZ::kan*) with natural transformation in the Stdys021 strain, although with transformation frequencies of 4.96 × 10^−5^ (*n* = 3, SD = 1.23 × 10^−5^).

Having confirmed that natural transformation is possible in Stdys021 (SDSD), we next wanted to test whether natural transformation is possible in other strains, including SDSE strains. We tested the same protocol to produce *lacZ* deletions in three additional strains having a complete set of competence genes (Fig. 2): Stdys042 (SDSD), iSDSE-NORM77 (SDSE), and iSDSE-NORM6 (SDSE). While no mutants were obtained for Stdys042, Δ*lacZ::kan* mutans were obtained in both iSDSE-NORM77 and iSDSE-NORM6 with average transformation rates of 2.71 × 10^−7^ (*n* = 3, SD = 9.36 × 10^−8^) and 9.06 × 10^−8^ (*n* = 3, SD = 1.28 × 10^−7^), respectively.

### Variations in lactose metabolism pathways as indicators of host-specific adaptations in *S. dysgalactiae*

**Fig 5:**
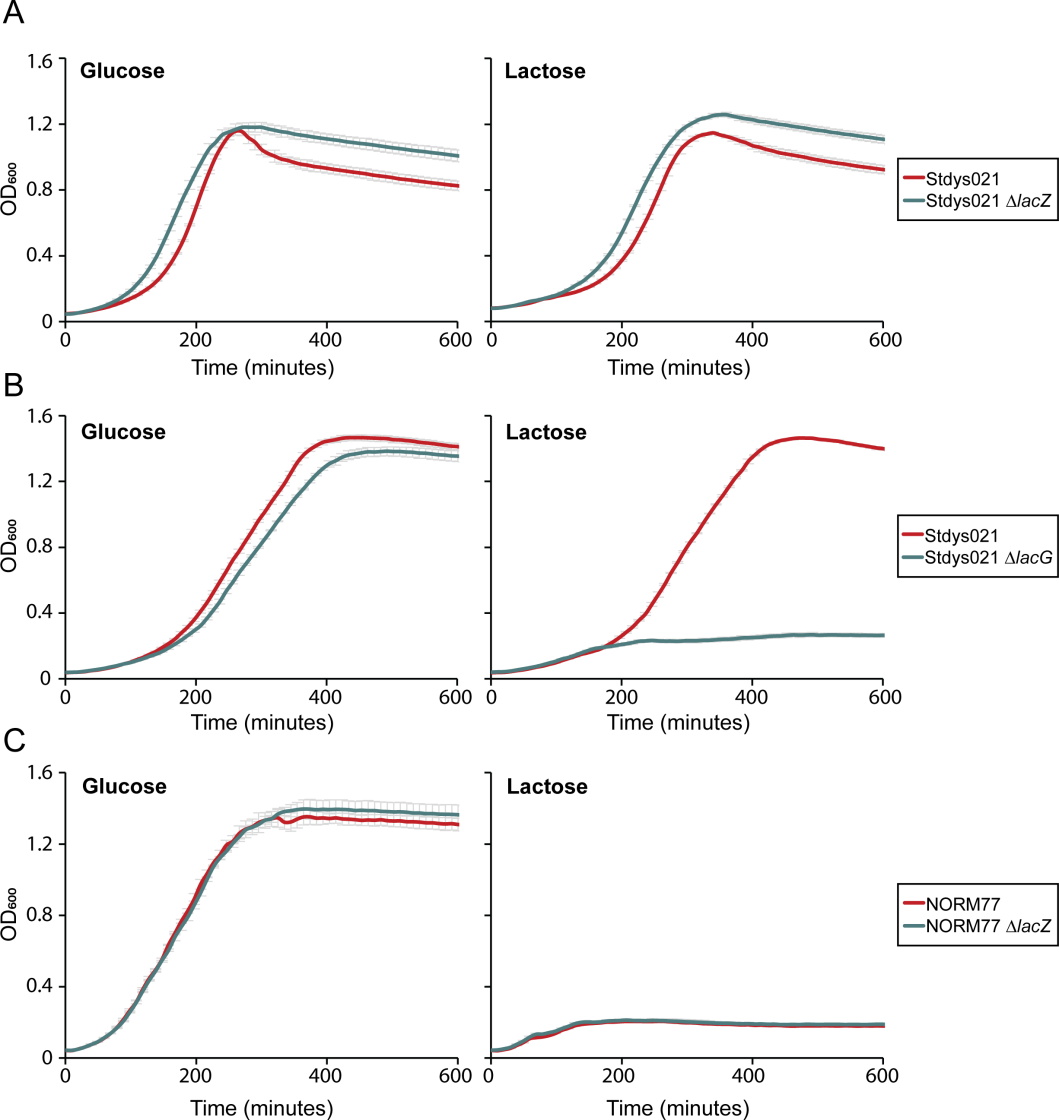
Analysis of genes involved in *S. dysgalactiae* growth on lactose. Growth curves of Stdys021 wild type and ∆*lacZ* (MM537) (**A**), Stdys021 wild type and ∆*lacG* (**B**) and iSDSE-NORM77 wild type and ∆*lacZ* (**C**) with 0.5% glucose or 0.5% lactose as carbon source in the C-medium. Strains were diluted 1/50 and grown to an OD_600_ of 0.4 before re-diluting to OD 0.05 in C-medium with either 0.5% glucose or 0.5% lactose as carbon source. Data are represented as mean values from three replicates, with error bars indicating standard deviations.

Having established a method to create gene knockouts in *S. dysgalactiae*, we next used this to explore adaptations to growth on lactose in this species. The *S. dysgalactiae* SDSD strain Stdys021 has been isolated from a bovine mastitis case and is thus likely to be adapted to growth in milk. It has previously been shown that bovine adapted isolates of *S. agalactiae* and *S. dysgalactiae* (SDSD) possess a lactose utilization operon (*lac2*), which provides a selective growth advantage in lactose-rich environment such as milk (35–37). This operon is, however, absent in iSDSE-NORM77. Previously, human-associated SDSE isolates have been found to harbor only a decayed *lac2* element (36). The *lac2* operon in Stdys021 encodes a phosphotransferase system for lactose uptake and a 6-phospho-β-galactosidase (*lacG*) for cleavage of lactose-6-phosphate to galactose-6-phosphate and glucose. Galactose-6-phosphate is further converted to glyceraldehyde-3-phosphate by LacA, LacB, LacC, and LacD through the tagatose-6-phosphate pathway (38). As noted above, both Stdys021 (SDSD) and iSDSE-NORM77 (SDSE) harbor another β-galactosidase, *lacZ*, whose role in lactose utilization has not been investigated in these species. To investigate the roles of *lacG* and *lacZ* in lactose metabolism in *S. dysgalactiae* subsp. *dysgalactiae*, we also deleted *lacG* in Stdys021 by allelic replacement, resulting in inactivation of the *lac2* operon, and assessed the ability of Δ*lacG* and Δ*lacZ* mutants to grow in C-medium with either glucose or lactose as carbon source (Fig. 5A and B). We found that LacZ is not required for growth on lactose in Stdys021; however, the knockout of *lacG* resulted in a significant reduction in growth. The SDSE strain iSDSE-NORM77, which does not harbor the *lac2* operon (and thus not *lacG*), displayed very limited growth on lactose under these conditions, and this was not further decreased in the *lacZ* deletion mutant (Fig. 5C). Together, this supports the notion that the 6-phospho-β-galactosidase LacG is the primary enzyme utilized for lactose fermentation in *S. dysgalactiae* and that the *lac2* operon plays a critical role in the adaptation of *S. dysgalactiae* (SDSD) to the bovine mammary gland.

## DISCUSSION

Natural transformation is an important mechanism for horizontal gene transfer in bacterial species across the phylogenetic tree (8) that has been widely exploited for genetic modification in different species, including various streptococci (9, 10, 13, 15–17). Although *S. pyogenes* has been observed to undergo natural transformation in biofilms on epithelial cells and during *in vivo* colonization in mice (30), there are, to our knowledge, no protocols allowing the use of natural transformation to make gene modifications of streptococci within the pyogenic group. In this work, based on genomic and transcriptional analysis, we have been able to utilize natural transformation to make gene knockouts by allelic replacement in *S. dysgalactiae* using linear DNA as transforming DNA. The results presented here thus allow for more efficient gene modification in this species and provide a platform for further development of such techniques for pyogenic streptococci.

While we successfully demonstrated the potential for making gene knockouts by natural transformation in *S. dysgalactiae*, our results suggest the presence of yet unidentified factors that may limit the transformation process. For instance, despite harboring a complete set of competence genes and lacking the known competence inhibitor paratox, we were unable to obtain mutants in one of the four strains tested (Stdys042). Our transcriptional reporter assays confirmed that expression from the P*_comX_* and P*_ssbB_* promoters was indeed induced in Styds042 upon XIP induction (Fig. S3), verifying that the transcriptional activation of the competence pathway is functional in this strain. This suggests that unknown factors downstream of competence activation might influence the natural transformation process.

From the reporter assays, it was noted that the expression from P*_comX_* and P*_ssbB_* (as a proxy for level of competence induction) varied between single cells in the population (Fig. 3). The single-cell heterogeneity is in line with previous observations in *S. mutans* (39). On the population level, the luciferase-based reporter assays and RNA-seq showed that the rapid expression of competence genes upon XIP exposure (Fig. 3A, B, and 4; Table 1; Tables S3 to S6) is followed by a long period of active expression, which has also been reported for *S. mutans* (13, 40). The expression patterns displayed considerable temporal fluctuations, which were different between strains (Fig. 3A and B). It remains to be determined whether the fluctuating expression patterns which emerged during the luciferase reporter assays are functionally relevant, but such phenomena have not yet been observed in other species. These findings further indicate that, while the competence genes are conserved across strains, their expression and, consequently, the induction of competence are subject to strain-specific regulatory influences. Variations in expression levels between strains may arise from differences in XIP-uptake, XIP-ComR-binding efficiency and specificity, or ComR-promoter interactions.

Moreover, the transformation efficiency was consistently lower than what has been reported for other species such as *S. mutans* (13) and *S. suis* (18), but similar to transformation rates found for *S. thermophilus* and *S. vestibularis* (19). We also noted that the transformation efficiencies were rather variable, and only small changes to the transformation protocol led to complete loss of transformation. Although the transformation efficiency may be intrinsically low in *S. dysgalactiae*, most likely, this phenomenon is caused by a combination of genetic and environmental factors affecting the competence regulation. In *S. mutans*, the regulation of competence by ComRS has been shown to involve a complex network of interactions between different two-component regulatory systems influenced by components in the growth medium [reviewed in Shanker and Federle (41)], and more work will be needed to decipher any such interaction in *S. dysgalactiae*. In this work, we chose to use C-medium, which has been shown to support competence development in *S. pneumoniae* (42), to optimize the protocol (see Results for details). Given the observed strain variability, further optimization of conditions in this protocol, including but not limited to more defined growth media, different incubation steps, and higher XIP concentrations, may be required when attempting to transform new strains.

Furthermore, *S. dysgalactiae* may have some species-specific properties related to early competence gene regulation; RNA-seq in iSDSE-NORM77 showed an early and sustained upregulation of *comS* in response to XIP but a delayed upregulation of *comR* (Table 1). Also, for Stdys021, only *comS* was upregulated at the first time point, while *comR* remained non-regulated after XIP addition in these experiments (Table 1). This difference in regulation may be explained by the *comR* promoter being devoid of any apparent ComR-XIP binding sites (P1 consensus pattern/Ecom-box), which is similar to, for example, *S. thermophilus* (24, 43). Expression of *comR* has instead been suggested to be regulated by ComX in *S. pyogenes* due to the presence of a CIN-box immediately upstream *comR* (44), but no CIN-box is located immediately upstream *comR* in *S. dysgalactiae*. In *S. mutans*, regulation of *comR* has been found to be partially dependent upon a CIN-box located within a gene, 580-bp upstream *comR* (44). Such a potential CIN-box (differing in two bases, GCCGAATA, from the consensus sequence) is also found in *S. dysgalactiae*, potentially giving rise to a delayed response to XIP in iSDSE-NORM77. In the RNA-seq experiment, the expression level of *comX* in Styds021 was elevated already prior to XIP induction, in contrast to iSDSE-NORM77, where *comX* was highly upregulated by XIP (Table 1). The high initial expression of *comX* in Stdys021 was unexpected, and in contrast to what was found in the reporter assays (Fig. 3), and we cannot fully explain this observation. Possibly, the difference between the reporter assays and the RNA-seq results could be caused by the slight differences in experimental setup and suggests a finely tuned regulation of competence in this species.

In this study, we have demonstrated the capacity of *S. dysgalactiae* for natural competence, facilitating DNA uptake and integration. This raises intriguing questions about the ecological and evolutionary roles of natural transformation within this species, including the specific environmental triggers that induce competence. While the exact conditions that trigger *S. dysgalactiae* to undergo natural transformation *in vivo* remain unidentified, studies in *S. pyogenes* suggest that competence may be induced in biofilms (30). Existing research highlights the critical role of recombination of small genomic fragments as a major driver for core genome diversity in SDSE (45, 46). Both intra-species and inter-species genetic exchanges have been documented, with significant evidence of horizontal gene transfer through homologous recombination between the core genomes of SDSE and *S. pyogenes* (45–47). Such lateral gene transfer, especially involving housekeeping genes and the *emm* gene, encoding the key M-protein virulence determinant, has been found to be important for driving genetic variability in the SDSE population (46–49). Specifically, the detection of identical *emm* genes in genetically diverse SDSE isolates have been reported, alongside observations of multiple *emm* genes within the same genetic background, and the acquisition of *S. pyogenes emm* genes in SDSE isolates (46, 50, 51). The exchange of accessory virulence or antimicrobial resistance genes is predominantly attributed to the exchange of mobile genetic elements, yet the mechanisms facilitating the transfer of core genes remain elusive (45). It could therefore be speculated that natural transformation plays a key role here alongside other mechanism of horizontal gene transfer.

The variability in the presence of competence genes is another interesting point in the understanding of the role of natural transformation in this species. Nearly half of the bovine-derived strains (SDSD) and 30% of the human-derived strains (SDSE) harbored non-intact core competence-regulated genes, in particular genes involved in DNA uptake, and therefore most likely lack the capability to perform natural transformation (Fig. 2). Interestingly, however, the regulatory genes *comR*, *comS*, and *comX* appear to be intact in all strains. It should be noted that 75% of the SDSD strains had a genetic configuration with an IS element located between the *comR* and *comS* genes. We performed reporter assays in such a strain (B237-3), which indicated that competence could be induced (Fig. S4). On the other hand, attempts to obtain transformants via knockout of the *lacG* gene in B237-3 were unsuccessful. The reason for the lack of transformation remains to be understood, but underlines strain-strain variation in transformation capacity between strains with different genetic configurations (Table S1). The retention of competence regulatory genes, even when the ability to perform natural genetic transformation is absent, might suggest that competence induction confer additional benefits beyond their role in DNA uptake. This is indeed in line with what has been suggested for other species, where competence has been linked to DNA repair, improved survival during exposure to antibiotics and other stressors, metabolic adaptations, or for expression of virulence traits (26, 52–57). Related to stress responses, competence has been proposed to function as a general stress response similar to the SOS response in *Escherichia coli* (33, 34, 57, 58). Our RNA-seq analysis indicates that the HrcA regulon, involved in modulating protein-folding and degradation pathways under heat stress, is upregulated during competence in *S. dysgalactiae*, suggesting that competence may be associated with a more limited stress response in this species.

In conclusion, our study provides novel insights into the regulation and potential implications of natural transformation in pyogenic group streptococci. While further work is needed to fully understand the conditions under which natural transformation occurs *in vivo* and the potential involvement of natural transformation in acquisition of new genetic traits in *S. dysgalactiae*, our findings lay the groundwork for these future investigations. Importantly, the ability of genetic manipulation by natural transformation opens new possibilities of functional genetics in *S. dysgalactiae*. We demonstrate that a bovine udder-derived SDSD can utilize lactose as the sole carbon source due to the presence of the *lac2* operon, which is conserved across all SDSD strains but decayed in SDSE isolates (36, 59, 60). This is the similar pattern observed in *S. agalactiae*, where the *lac2* operon predominates in bovine-associated strains and is linked to lactose metabolism, suggesting a shared evolutionary pathway among *Streptococcus* spp. adapted to the lactose-rich environment of the bovine mammary gland (35). This exemplified the power of using natural transformation to generate mutants for studying molecular mechanisms underlying its virulence and host adaptation in these species.

## MATERIALS AND METHODS

### Bacterial strains, plasmids, and growth conditions

The bacterial strains and plasmids used in the present study are listed in Table S7. *E. coli* strains were grown in lysogeny broth or on lysogeny agar supplemented with spectinomycin (60 µg/mL) for selection. *S. dysgalactiae* was grown in airtight tubes at 37°C in brain heart infusion (BHI) or in C-medium (61) or on BHI agar at 37°C with 10% CO_2_. When required, spectinomycin (100 µg/mL), kanamycin (400 µg/mL), or streptomycin (200 µg/mL) was added to the media.

### Genome analysis of genes associated with competence and natural transformation

A collection of previously characterized SDSD and SDSE genomes derived from epidemiological surveillance studies in Norway (36, 62) was retrieved from GenBank. The genomes were screened for the presence of genes associated with competence and natural transformation (Table S1) using the BLASTn algorithm in Geneious Prime v.2023.0.4 (63). Query sequences were retrieved from *S. dysgalactiae* NCTC13759. The genetic context was manually inspected for mutations or insertion of mobile genetic elements resulting in truncated competence proteins, as well as mobile genetic elements inserted immediately upstream or downstream of competence genes. Insertion sequences, integrative conjugative elements, and bacteriophages were identified using ISfinder, ICEberg v.3.0, and Phastest, respectively (64–66).

### Construction of reporter strains

The P*_comX_*-*luc*-*gfp* and P*_ssbB_*-*luc*-*gfp* reporter plasmids were constructed by first amplifying the promoter regions of *comX* and *ssbB* from strain *S. dysgalactiae* iSDSE-NORM37 using primers mm53/mm54 and mm58/mm59, respectively. Overlap sequences complementary to the *luc-gfp* fragment and restriction sites were introduced with the primers. The *luc*-*gfp* fragment was amplified from strain *S. pneumoniae* MK175 using primers mm55/mm57. P*_comX_* and P*_ssbB_* were fused with *luc*-*gfp* by overlap extension PCR using primers mm53/mm57 and mm58/mm57, respectively. The resulting promoter-*luc*-*gfp* fragment was digested with restriction enzymes SalI-HF and NheI-HF and ligated into the SpeI-HF and SalI-HF of pFD116 (67). Enzymes were purchased from New England Biolabs. The ligation was transformed into *E. coli* DH5α with spectinomycin selection, and correct construct was verified by sequencing.

Electroporation of plasmids into *S. dysgalactiae* strains was performed based on previous protocols (68, 69). Cells were grown overnight at 37°C in C-medium. The overnight cultures were diluted 1/100 in the same medium and incubated at 37°C until an OD_600_ of 0.4 was reached. Cells were centrifuged at 4,000 × *g* for 10 min at 4°C. The pellet was washed four times in 10% ice-cold glycerol. Cells were re-suspended in 2–3 mL 10% glycerol with 0.5 M sucrose. Electrocompetent cells were stored at −80°C. Electroporation was carried out using a MicroPulser (BiorRad) at 2.1 V, 100 Ω, and 25 µF (68). Plasmid DNA (500–1,000 ng) was added to 50 µL of competent cells and placed in a 1-mm electroporation cuvette. Immediately after electroporation, 950 µL recovery medium (TSB with 0.5 M sucrose) was added, and cells were incubated at 37°C for 2 hours prior to plating onto BHI agar containing appropriate antibiotics.

### Synthetic peptides

Synthetic peptides (purchased from Thermo Scientific) were dissolved in dH_2_O (XIP2, QVDWWRL) or DMSO (XIP1, EFDWWNLG).

### Luciferase reporter assays

Overnight cultures of reporter strains were diluted 1/50 in C-medium in closed screw-cap tubes and grown at 37°C until mid-logarithmic phase. Cultures were diluted to an OD_600_ of 0.01 in C-medium with 170-µg/mL D-luciferin (Invitrogen). Cultures were grown at 37°C in a Hidex Sense microplate reader in volumes of 300 µL in white flat-bottom 96-well plates (Corning). OD_600_ and luminescence were measured every 5th min. When appropriate, 250-ng/mL XIP was added at OD_600_ of 0.1. A 300 µL aliquot of cell-free culture medium with D-luciferin served as background control.

### Phase contrast and fluorescence microscopy analysis

To monitor competence development at single cell level, overnight cultures of reporter strains were diluted 1/50 in C-medium in closed screw-cap tubes and grown at 37°C until mid-logarithmic phase. Cultures were diluted to an OD_600_ of 0.01 in 50 mL C-medium. When cultures reached an OD_600_ of 0.1, samples were taken for microscopy, while the remaining culture was divided into two aliquots of 14 mL. One of the aliquots was induced to competence by adding 250 ng/mL XIP. Samples were incubated further for 1 hour at 37°C before taking samples for microscopy. All samples were done in triplicate. Microscopy was performed on a Zeiss AxioObserver with ZEN Blue Software. Images were captured with an ORCA-Flash v.4.0, V2 Digital CMOS camera (Hamamatsu Photonics) through a ×100 PC objective. For fluorescence microscopy, HPX 120 Illuminator (Zeiss) was used as a light source. Image analysis was performed using MicrobeJ (70), and plotting was done in Rstudio.

### RNA isolation and RNA sequencing

Cell cultures grown to an OD_600_ of 0.5 were re-diluted to an OD_600_ of 0.01 in 100 mL C-medium in a screw-cap flask and incubated at 37°C. When OD_600_ reached 0.1, 10 mL culture was withdrawn to harvest cells, while the remaining culture was induced to competence by adding 250 ng/mL XIP and immediately divided into 10-mL aliquots, designated for harvesting at specific time points post XIP induction. The cultures were incubated further at 37°C until the designated harvesting times (either 10 and 60 min post-XIP addition for iSDSE-NORM77 or 5 and 30 min post-XIP addition for Stdys021). Cells were harvested by centrifugation at 6,000 × *g* for 1 min at 4°C. The pelleted cells were immediately frozen in liquid nitrogen and stored at −80°C for subsequent RNA extraction.

RNA was isolated using the RNeasy mini kit (Qiagen), followed by DNase treatment and phenol chloroform extraction as previously described in Stamsås et al. (71). Library construction, quality control, and sequencing were performed at Novogene (UK). Briefly, rRNA was depleted from total RNA, and second-strand cDNA synthesis was conducted using dUTPs instead of dTTPs to create a directional library (PE150). Quantified libraries were pooled and sequenced on Illumina platforms. After quality check, the reads were aligned against *S. dysgalactiae* iSDSE-NORM77 (ERR10679354) or *S. dysgalactiae* Stdys021 (ERS5685029), and expression levels were calculated using Geneious v.2023.0.4 (63). The genomes of Stdys021 and iSDSE-NORM77 were annotated using the Prokka pipeline (Galaxy v.1.14.6+galaxy1) (72, 73). Differential gene expression analysis was performed with Geneious v.2023.0.4 using the median of gene expression ratios for normalization of transcripts. Duplicate samples were analyzed for Stdy021, while one replicate was analyzed for iSDSE-NORM77.

### *S. dysgalactiae* transformation protocol and construction of mutants

Unless designated otherwise, overnight cultures were diluted to an OD_600_ of 0.05 in C-medium and grown at 37°C in closed screw-cap tubes until an OD_600_ of 0.1–0.2 was reached. Cultures were re-diluted to an OD_600_ of 0.02–0.03 and incubated further at 37°C. When cultures reached an OD_600_ of 0.05, ~500 ng DNA harboring an antibiotic resistance marker (linear PCR product of 5.3 kb consisting of the resistance cassette flanked by 2-kb homology regions) and 250 ng/mL XIP was added to 1 mL culture in an Eppendorf tube. Cultures were incubated further at 37°C for 3–4 hours. Cells were plated on BHI agar containing 400 µg/mL kanamycin for selection of transformants and incubated at 37°C in 10% CO_2_ for 24 hours. Transformation frequency was calculated as the ratio of transformants (kanamycin-resistant colony-forming units [CFU]) to the total CFU count per 1 µg/mL of DNA.

To create genetic knockouts, the ~2,000-bp regions upstream and downstream of the target gene were fused to the 5′ and 3′ end, respectively, of a kanamycin resistance cassette by overlap extension PCR (74). All primers used in this work are listed in Table S8. To construct *lacZ* knockout mutants, upstream and downstream *lacZ* was amplified with primers mm143/mm144 and mm145/mm146 for Styds021, and with primers mm143/mm144 and mm145/mm179 for iSDSE-NORM6 and iSDSE-NORM77. To construct *lacG* knockout mutants, upstream and downstream *lacG* was amplified with primers mm165/mm166 and mm168/mm170 for Stdys021. The amplicons were transformed into *S. dysgalactiae* to knock out the target gene by homologous recombination using the protocol for transformation described above. Knockout mutants were screened by PCR to verify correct insertion and absence of deleted gene.

### Growth assays

For examining growth in liquid cultures, overnight cultures were diluted 1/50 in C-medium and grown to an OD_600_ of ~0.4. Cultures were then diluted to an OD_600_ of 0.05 in C-medium with either 0.5% glucose or 0.5% lactose as carbon source. Growth of cultures (300 µL in a flat-bottom 96-well plate [Sarstedt]) was monitored at 37°C in a Hidex Sense microplate reader, measuring OD_600_ every 10 min.

### Quantification of organic acids, carbohydrates, and volatile compounds using chromatography

Organic acids and carbohydrates were analyzed using HPLC as described by Grønnevik et al. (75). One gram of liquid sample was added to a glass tube and mixed with 2.5 mL mqH_2_O, 0.2 mL 0.5 M H_2_SO_4_ (Merck), and 8 mL acetonitrile (Merck). For the remaining part, the protocol from Grønnevik et al. (75) was followed. Standard solutions for external calibration were prepared the same way as the samples, and organic acids and carbohydrates were identified according to their retention time compared with the standard solutions. The organic acids used for standard solutions were pyruvic acid, lactic acid, acetic acid, and DL-pyroglutamic acid (Sigma-Aldrich), and the carbohydrate was glucose (Merck).

Volatile compounds were measured using HSGC as described (75). Helium was used as carrier gas at a flow of 11.1 mL/min. For the remaining part, the protocol was followed. Peaks were identified according to their retention times and quantified using external standard solutions of acetaldehyde (Sigma-Aldrich) and ethanol (Merck).

## Data Availability

The raw FASTQ data are accessible at https://www.ebi.ac.uk/ena/browser/home with accession number PRJEB74091.

## SUPPLEMENTAL MATERIAL

Supplemental Material msphere.00214-24-s0001.pdf Fig. S1-S4; Tables S7 and S8.

Supplemental Table msphere.00214-24-s0002.xlsx Table S1.

Supplemental Tables msphere.00214-24-s0003.xlsx Tables S2-S6.

ASM does not own the copyrights to Supplemental Material that may be linked to, or accessed through, an article. The authors have granted ASM a non-exclusive, world-wide license to publish the Supplemental Material files. Please contact the corresponding author directly for reuse.
