# Supplementary material for: Genetic modification of Streptococcus dysgalactiae by natural transformation
Source: mSphere. 2024 Jun 21;9(7):e00214-24. doi: 10.1128/msphere.00214-24 (PMC11288034; doi:10.1128/msphere.00214-24)
Supplement: Supplemental Material — Fig. S1-S4; Tables S7 and S8. [file msphere.00214-24-s0001.pdf]

Supplementary material

Genetic modification of *Streptococcus dysgalactiae*

by natural transformation

Marita Torrissen Mårli, Oddvar Oppegaard, Davide Porcellato, Daniel Straume, Morten Kjos

Supplementary figures

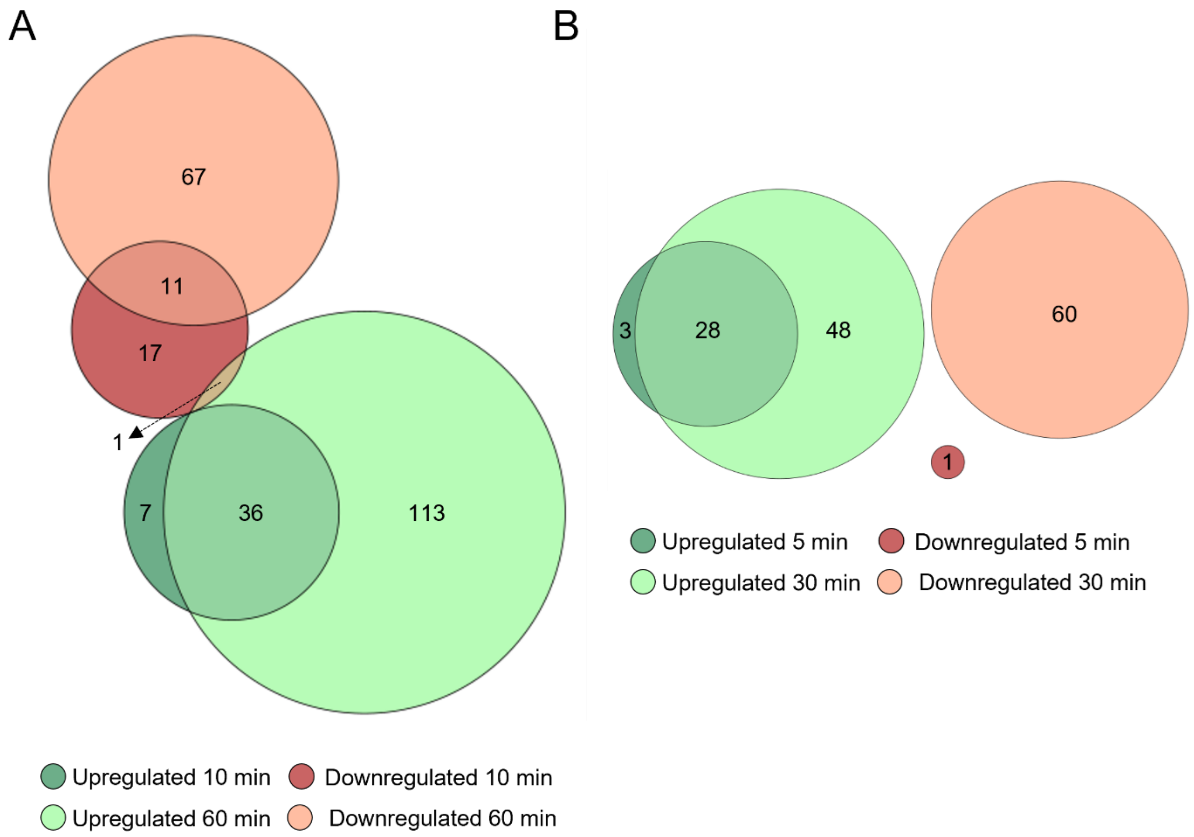

Fig. S1. (A) Venn diagram of differentially expressed genes in NORM77. Diagrams show how many genes were upregulated (green) or downregulated (red) at 10 or 60 minutes, using a cutoff log2 fold change value of 1. Venn diagrams were created using http://eulerr.co. (B) Venn diagram of differentially expressed genes in Stdys021. Diagrams show how many genes were upregulated (green) or downregulated (red) at 5 or 30 minutes, using a cutoff log2 fold change value of 1. Venn diagrams were created using http://eulerr.co.

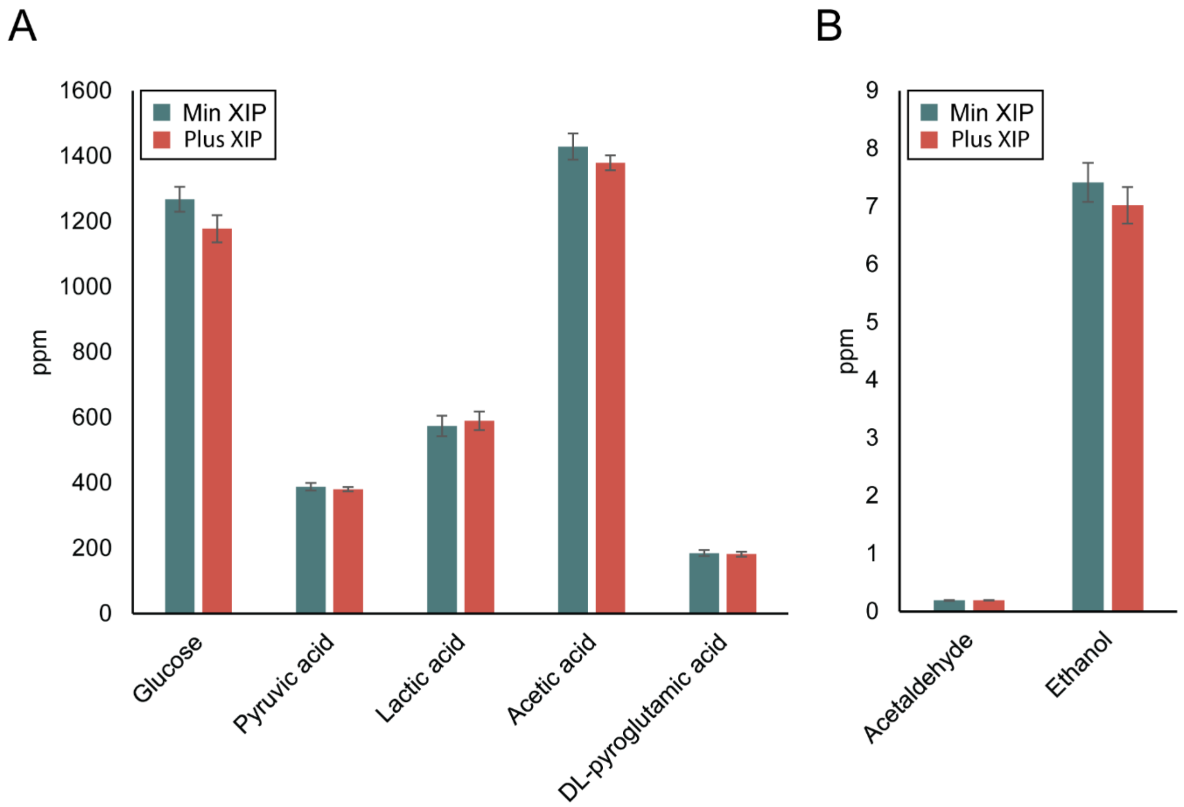

Fig. S2 Analysis of metabolite levels in competent *S. dysgalactiae*. Filter-sterilized supernatants from cultures one hour after induction to competence were analyzed by **(A)** high-Performance Liquid Chromatography (HPLC) for analysis of glucose, pyruvic acid, lactic acid, acetic acid and DL-pyroglutamic acid, and **(B)** headspace Gas Chromatography (HSGC) for analysis of acetaldehyde and ethanol. Cultures were induced to competence by addition of 250 ng/ml XIP2 at an OD_{600} of 0.1. Data is represented as mean values from three biological replicates, with error bars indicating standard deviation.

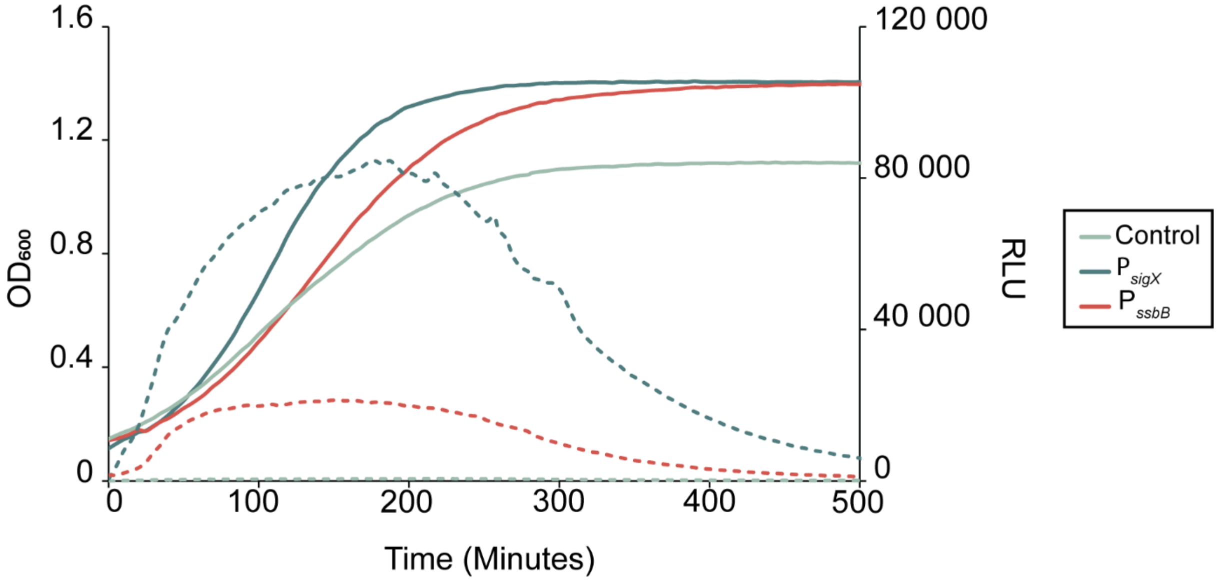

Fig. S3 Luciferase expression from the P*comX*-*luc*-*gfp* and P*ssbB*-*luc*-*gfp* reporters in *S. dysgalactiae* Stdys042 after addition of XIP. The cells were diluted to an OD_{600} of 0.01 and grown at 37 °C until an OD_{600} of 0.1, at which time 250 ng/ml XIP was added. OD_{600} and luminescence (as RLU, relative luminescence units) were measured every 5 minutes. Control strain harbors the pFD116 vector. Solid lines indicate growth (OD_{600}), while dashed lines correspond to RLU.

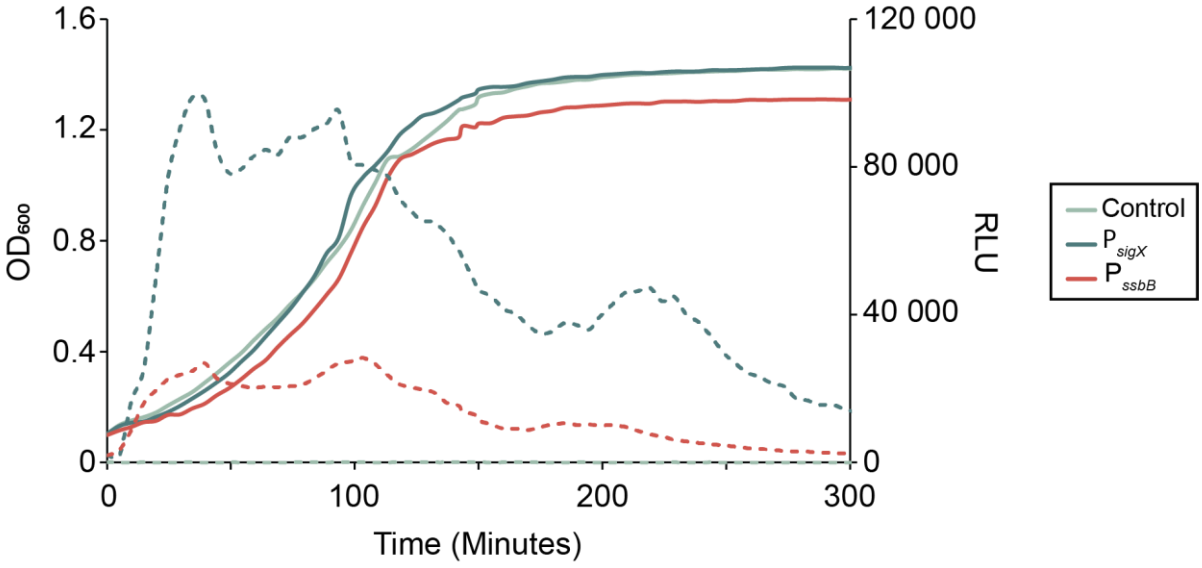

Fig. S4. Luciferase expression from the P*comX*-*luc*-*gfp* and P*ssbB*-*luc*-*gfp* reporters in *S. dysgalactiae* B237-3 (strain with an IS-element between *comR* and *comS*) after addition of XIP. The cells were diluted to an OD_{600} of 0.01 and grown at 37 °C until an OD_{600} of 0.1, at which time 250 ng/ml XIP was added. OD_{600} and luminescence (as RLU, relative luminescence units) were measured every 5 minutes. Control strain harbors the pFD116 vector. Solid lines indicate growth (OD_{600}), while dashed lines correspond to RLU.

Supplementary tables

Table S1. Overview of the presence of intact or inactive competence and natural transformation genes within *S. dysgalactiae*.

Table S2. Expression values in transcript per million (TMP) from RNA-seq of all NORM77 and Stdys021 genes. Time-points are indicted (t0, t10, t60, t5, t30). Results from the two parallel experiments in Stdys021 are shown. Locus tags, gene names and gene functions are given.

Table S3. Differential expression of all NORM77 genes. Differential expression is given as log2 fold change (L2FC) at time points 10 and 60 minutes after addition of XIP compared to cells immediately prior to XIP addition (0 minutes). Upregulation (L2FC >1) is highlighted in green and downregulation (L2FC<1) in red.

Table S4. Differential expression of all Stdys021 genes. Differential expression is given as log2 fold change (L2FC) at time points 5 and 30 minutes after addition of XIP compared to cells immediately prior to XIP addition (0 minutes). Upregulation (L2FC >1) is highlighted in green and downregulation (L2FC<1) in red. Data from two parallel experiments are shown in the table.

Table S5. Differentially expressed genes in response to XIP addition in NORM77. Genes that were differentially regulated for at least one time point are listed. Upregulation (L2FC >1) is highlighted in green and downregulation (L2FC<1) in red. Data from two parallel experiments are shown in the table.

Table S6. Differentially expressed genes in response to XIP addition in Stdys021. Genes that were differentially regulated for at least one time point are listed. Upregulation (L2FC >1) is highlighted in green and downregulation (L2FC<1) in red. Data from two parallel experiments are shown in the table.

Table S7 Strains used in this study.

Strain	Genotype and characteristics[a]	Reference
E. coli		
DH5α	Cloning host	Lab collection
MM212	DH5α, pFD116	This study
MM240	DH5α, pFD116-P*comX-luc-gfp*, spcR	This study
MM241	DH5α, pFD116-P*ssbB-luc-gfp*, spcR	This study
S. dysgalactiae		
Stdys021	SDSD-isolate	(1)
Stdys042	SDSD-isolate	(1)
NORM6	SDSE-isolate	(2)
NORM77	SDSE-isolate	(2)
NORM37	SDSE-isolate	(2)
B237-3	SDSD-isolate	(1)
MM413	NORM77, pFD116, spcR	This study
MM414	NORM77, pFD116-P*comX-luc-gfp*, spcR	This study
MM415	NORM77, pFD116-P*ssbB-luc-gfp*, spcR	This study
MM417	Stdys021, pFD116, spcR	This study
5MM418	Stdys021, pFD116-P*comX-luc-gfp*, spcR	This study
MM419	Stdys021, pFD116-P*ssbB-luc-gfp*, spcR	This study
MM496	B237-3, pFD116, spcR	This study
MM497	B237-3, pFD116-P*comX-luc-gfp*, spcR	This study
MM498	B237-3, pFD116-P*ssbB-luc-gfp*, spcR	This study
MM519	Stdys021, Δ*lacG::kan*, kanR	This study
MM537	Stdys021, Δ*lacZ::kan*, kanR	This study
MM538	NORM6, Δ*lacZ::kan*, kanR	This study
MM539	NORM77, Δ*lacZ::kan*, kanR	This study
S. pneumoniae		
ADP62	ΔbgaA::P*ssbB-luc-gfp* , Δ*comA::ery*	(3)

[a] spcR = spectinomycin resistant, kanR = kanamycin resistant

Table S8. Oligos used in this study.

**Oligo name**	**Sequence (5' – 3')**
**Primers for construction of luc-gfp reporter plasmids**	
mm53_PsigX_NheI_F	GTGTCTGCTAGCGAAGAGTGTGGTAAGATAGAG
mm54_PsigX_over_luc_R	TTATGTTTTTGGCGGATCTCATCATGATTCTCCTTTTCTTTTAAAC
mm55_luc_F	ATGAGATCCGCCAAAAACATAAAG
mm57_luc_gfp_SalI_R	TGCTTCGTCGACGAATCTTGCTTGGCAAGGTTC
mm58_PssbB_NheI_F	GTGTCTGCTAGCTAGATGTTCGGCAGTCAGAC
mm59_PssbB_over_luc_R	TTATGTTTTTGGCGGATCTCATATCAACCTCCTTACCTTATTATTC
**Primers for amplification of kanamycin resistance cassette**	
Kan484F	GTTTGATTTTTAATGGATAATGTG (4)
RpsL41R	CTTTCCTTATGCTTTTGGAC (4)
**Primers for construction of Δ*lacZ*::kan**	
mm143_up_lacZ_F	CCTTTGCGAGTCTGCTTCG
mm144_up_lacZ_over_janus_R	CACATTATCCATTAAAAATCAAACATCAGTCTCCTGCTCCTCG
mm145_down_lacZ_over_janus_F	GTCCAAAAGCATAAGGAAAGATCAAGTCAACCTCTGTTAAGG
mm146_down_lacZ_R	TGCTATTGAATCGCTCTGCC
mm179_down_lacZ_SDSE_R	TGCTGGTAGAGCACTCTCG
**Primers for construction of Δ*lacG*::kan**	
mm165_up_lacG_F	GTAGTGAATGCTTGAACGATG
mm166_up_lacG_over_janus_R	CACATTATCCATTAAAAATCAAACCCTCTTCTAAATTTTCATTTCA C
mm168_down_lacG_over_janus_F	CTCGAGCGGCCGCATAGTGTAGCAGAAACTCAAGTGATTG
mm170_down_lacG_R	ATCTGCCCACTCAGCTATAC

REFERENCES

1. Porcellato D, Smistad M, Skeie SB, Jørgensen HJ, Austbø L, Oppegaard O. 2021. Whole genome sequencing reveals possible host species adaptation of *Streptococcus dysgalactiae*. Sci Rep 11:17350.
2. Kaci A, Jonassen CM, Skrede S, Sivertsen A, Steinbakk M, Oppegaard O. 2023. Genomic epidemiology of *Streptococcus dysgalactiae* subsp. *equisimilis* strains causing invasive disease in Norway during 2018. Front Microbiol 14:1171913.
3. Moreno-Gámez S, Sorg RA, Domenech A, Kjos M, Weissing FJ, van Doorn GS, Veening J-W. 2017. Quorum sensing integrates environmental cues, cell density and cell history to control bacterial competence. Nature Communications 8:854.
4. Johnsborg O, Eldholm V, Bjørnstad ML, Håvarstein LS. 2008. A predatory mechanism dramatically increases the efficiency of lateral gene transfer in *Streptococcus pneumoniae* and related commensal species. Mol Microbiol 69:245-253.
